# Mycotic Giant Cell Epitheliomatous Inverted Papilloma of the Gingiva

**DOI:** 10.4103/0974-777X.62871

**Published:** 2010

**Authors:** Naim Mohammed, Amit Kumar, John Vanessa T, Ahmad Syed S

**Affiliations:** *Departments of Pathology JNMC, and Maxillofacial Surgery ZADC, AMU, Aligarh, UP, India*

**Keywords:** Epithelioma, Giant cell, Gingival, Mycosis

## Abstract

This report presents histopathological evidence of mycotic infection of the gingival epithelium followed by inverted verrucous giant cell epitheliomata of the gingiva. Sections from biopsy tissue revealed intercellular spaces of the parabasal squamous epithelium parasitized by Periodic Acid Schiff stain positive branching septate hyphae and conidia of morphological appearance of Trichophyton. Epithelial cells presented epitheliomatous proliferation with formation of giant cells showing phagocytosed fragments of mold.

## INTRODUCTION

Some fungi are known for carcinogenic metabolites and signaling.[1] Dermatophytes are increasingly under study to explore their role in etiopathogenesis of granulomatous immune disease and neoplasm.[1,2] Presently, we discuss, in a case, pictorial evidence of inverted papillary giant cell epitheliomata with trichophyton infection of the gingival mucosa.

## SHORT HISTORY

A 52-year-old goldsmith's ash washerman with a history of extraction of aching right upper second molar tooth 2 years back followed by painful on-and-off swelling, managed by the patient by frequenting dentists' prescriptions and consumption of countryside liquor a bottle a day, was referred to this hospital. Gingival biopsy with clinical diagnosis of leukoplakia/squamous carcinoma was submitted for histopathological diagnosis. The patient had no history of fungal infection of the skin or nails. The total leucocyte count was 8,000 cells/mm^3^, differential leucocyte count - P_68_L_29_E_2_M_1_, fasting blood sugar 95 mg/dL, serum urea 29 mg/dL and serum creatinine 1.1 mg/dL. Screening tests for human immunodeficiency virus, hepatitis and syphilis were negative.

## MICROPHOTOGRAPHS

**Figure 1:**
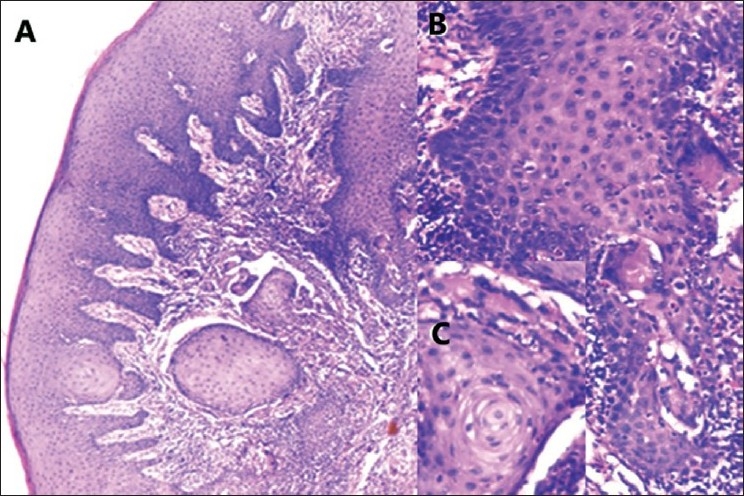
(A) Dyskeratosis, keratinocytic hyperplasia, parabasal epitheliomatous change and carcinoma cells infiltrating connective tissue [Hematoxylin and Eosin (H and E), ×125] and (B and C) pearl arrangement of keratinocytes in papillary core and epitheliomatous giant cells dotting the periphery (H and E, ×500)

Sections from the biopsy tissue revealed surface dyskeratosis with underlying keratinocytic hyperplasia of the gingival epithelium. Basal and parabasal layers presented with epitheliomatous proliferation of cells with formation of giant cells. Retepegs were broadened and elongated with keratinocytes in a pearl arrangement forming the core and hyperplastic cells dotted by giant cells at the periphery [Figure 1]. At places, well to moderately differentiated squamous cells were seen in the parabasal layer and microinfiltrating the underlying connective tissue.

**Figure 2:**
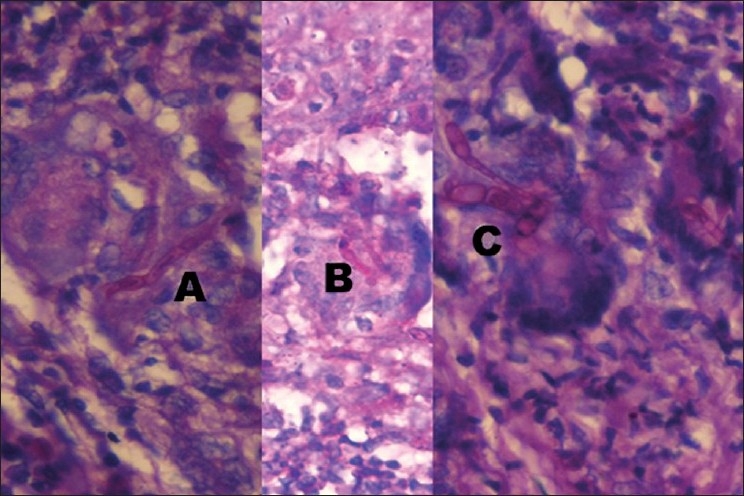
(A) PAS-positive hyphae parasitizing intercellular spaces between parabasal cells undergoing giant cell transformation (PAS, ×1,250), (B) phagocytosed fragments of septate fungal hyphae inside the parabasal epitheliomatous giant cell (PAS, ×1,250), and (C) conidia of trichophyton in the vicinity of epitheliomatous giant cells (PAS, ×1,250)

Periodic Acid Schiff (PAS) stained sections revealed septate branching fungal hyphae of mold parasitizing intercellular clefts in basal and parabasal squamous layers, with the cells around undergoing immunoactive and giant cell transformations [Figure 2A]. Some of the giant cells showed phagocytosed hyphae [Figure 2B]. Rarely, conidia characteristic of tricophyton were observed in close vicinity or within the giant cells [Figure 2C]. Above the parabasal layers, PAS-positive dust was seen in the keratinocytes, but no fragments of mold could be identified.

## DIFFERENTIAL DIAGNOSIS

Low-mitotic basal–parabasal epitheliomatous proliferation with dyskeratotic–acanthocytic hyperplasia, pearl formation and papillary clubbing and elongation of retepegs are the features diagnostic of epitheliomatous-inverted papilloma or verrucous–hyperplastic leukoplakia,[3] which differentiate this lesion from basal and squamous cell neoplasia. About 5% cases present with foci of carcinomatous change. Presence of giant cells formed by the fusion of basal–parabasal cells, with or without outpouching of the basal lamina, maintaining continuity with epitheliomatous cells and absence of connective tissue, qualifies the lesion as giant cell epithelioma/carcinoma[4,5] and distinguishes it from other granulomatous diseases like tuberculosis and sarcoidosis. Such a lesion is often caused by chronic irritation due to prolonged tobacco chewing or other microbial infections like human papillomavirus. Presently, trichophyton was identified as a cause of irritation and disease. Hyphae and conidia of the mold in histopathological sections help in identification of the fungus and differentiation from other granulomatous diseases. Giant cells in epithelioma call for detailed meticulous histopathological examination for the pathogenic organism.

## DISCUSSION

Histopathological evidence in the present case was diagnostic of mycotic giant cell epitheliomatous inverted papilloma of the gingiva. Oropharyngeal proliferative verrucous leukoplakia and its related lesions,[3] like inverted papilloma,[3] giant cell epitheliomata[4] and inverted verrucous carcinoma with or without epithelial giant cell formations,[5] have been known since the early 20^th^ century. Oral mycosis, particularly periodontal infection caused by dermatophytosis, and host-defensive activity of human cells in response to trichophyton have also been reported,[6,7] besides reports of fungal metabolites being responsible for granulomatous disease and cancer signaling.[1,2] Trichophyton infection with inverted verrucous giant cell epithelioma of the gingiva with squamous carcinomatous change, as presently observed, was not reported in the preexisting literature.

Dermatophytes are known for cutaneous infections and for invading and parasitizing superficial keratinocytic layers, while keratin is believed to be the source of nourishment for the mold.[1] In the present case, however, no identifiable fragment of mold could be found in keratinocytic areas. This could suggest either high immunocompetence of the keratinocytes or protective effect of saliva or of some unknown biological factor.

Dermatophytes are rarely known to infect the wet epithelium of the gum, except through periodontal mycosis leading to gingival hyperplasia.[6] History of toothache and extraction followed by a persistent problem in the present case also suggested the periodontal route, facilitating the mold to reach the parabasal layer of the gingival epithelium to invade and find a bioenvironment favorable for its survival. The mold appeared to act strategically, infecting and parasitizing younger and innocent parabasal acanthocytes, which, however, responded defensively by immunoactive giant cell epitheliomatous transformation. Histopathological evidences in the present case were affirmative of the view of mycotic granulomatous disease leading to neoplasm.[2]

## CONCLUSIONS

- Trichophyton mold can infest and parasitize the basal and parabasal layers of the gingival squamous epithelium. The portal of entry may be periodontal.
- Acanthocytes around the hyphae of the mold may respond by giant cell epitheliomatous response and verrucous keratinocytosis.
- Intercellularly parasitizing PAS-positive hyphae, phagocytosed segments of hyphae and conidia may be demonstrable in the giant cell epitheliomatous areas.
- No fungal hyphae or identifiable fragments of mold may be visible in areas of the gingival epithelium undergoing advanced keratinocytic change.
- Giant cells in the epithelioma call for detailed meticulous histopathological examination for the pathogenic organism.
